# Rationales, design and recruitment of the Taizhou Longitudinal Study

**DOI:** 10.1186/1471-2458-9-223

**Published:** 2009-07-10

**Authors:** Xiaofeng Wang, Ming Lu, Ji Qian, Yajun Yang, Shilin Li, Daru Lu, Shunzhang Yu, Wei Meng, Weimin Ye, Li Jin

**Affiliations:** 1 State Key Laboratory of Genetic Engineering and MOE Key Laboratory of Contemporary Anthropology, School of Life Sciences and Institutes of Biomedical Sciences, Fudan University, Shanghai, 200433, PR China; 2 CMC Institute of Health Sciences, Taizhou 225300, Jiangsu Province, PR China; 3 Clinical Epidemiology Unit, Qilu Hospital of Shandong University, Jinan 250000, PR China; 4 Institute of Public Health, Fudan University, Shanghai, PR China; 5 Department of Medical Epidemiology and Biostatistics, Karolinska Institutet, Stockholm, Sweden

## Abstract

**Background:**

Rapid economic growth in China in the past decades has been accompanied by dramatic changes in lifestyle and environmental exposures. The burdens of non-communicable diseases, such as cardiovascular diseases, diabetes and cancer, have also increased substantially.

**Methods/design:**

We initiated a large prospective cohort–the Taizhou Longitudinal Study–in Taizhou (a medium-size city in China) to explore the environmental and genetic risk factors for common non-communicable diseases. The sample size of the cohort will be at least 100,000 adults aged 30–80 years drawn from the general residents of the districts of Hailin, Gaogang, and Taixing (sample frame, 1.8 million) of Taizhou. A three-stage stratified sampling method will be applied. Baseline investigations include interviewer-administered questionnaire, anthropometric measurements, and collection of buccal mucosal cells and blood specimens. DNA will be extracted for genetic studies and serum samples will be used for biochemical examinations. A follow-up survey will be conducted every three years to obtain information on disease occurrence and information on selected lifestyle exposures. Study participants will be followed-up indefinitely by using a chronic disease register system for morbidity and cause-specific mortality. Information on non-fatal events will be obtained for certain major categories of disease (e.g., cancer, stroke, myocardial infarction) through established registry systems.

**Discussion:**

The Taizhou Longitudinal Study will provide a good basis for exploring the roles of many important environmental factors (especially those concomitant with the economic transformation in China) for common chronic diseases, solely or via interaction with genetic factors.

## Background

After years of state control of all productive assets, the Chinese government embarked on a major program of economic reform in 1978. Since then, China has made great progress in reducing the number of people living in poverty from 250 million at the start of its reform process in 1978 to 29 million in 2001 [1]. The living standards of the general population are far higher than ever before. Dramatic transformations such as urbanization, aging, westernization of diet and lifestyle, pollution (air, water, soil), city noise, stress and tension have simultaneously accompanied economic growth.

As a result of economic growth and associated sociodemographic changes, the burden from infectious diseases has gradually diminished, but the burden from cardiovascular diseases, cancer, diabetes mellitus, and other chronic diseases has increased substantially. Chronic, non-communicable diseases now account for an estimated 80% of total deaths and 70% of disability-adjusted life-years (DALYs) lost in China [2]. For cancers alone, the absolute numbers of deaths from all cancers increased from 1.2 million in 1991 to 1.5 million in 2000, and 1.8 million in 2005. For cardiovascular disease alone, people aged 35–64 years lost 6.7 million years of productive life during year 2000 at a cost to the country of around US$ 30 billion [3].

Some risk factors are known to have contributed to the prevalence of non-communicable chronic diseases in China. In 2000, 60.2% (147 million) of men and 6.9% (16 million) of women aged 35–74 years in China were current smokers [4]. Findings from the 2002 National Nutrition and Health Survey (NNHS) indicate that about 18% of Chinese adults are hypertensive, which equates to 153 million individuals [5]. Data from the 2002 NNHS showed that there are 184 million overweight people and a further 31 million obese people in China. The number of overweight and obese individuals is increasing rapidly, especially among children [6]. Westernization of lifestyle may also contribute to this scenario. Today, supported by generous pensions, most salaried persons can enjoy their leisure time. Enjoyment of delicacies and sedentary entertainment such as playing Mahjong and watching television are examples of activities which people spend during their leisure time in China, and which may lead to an increased risk of chronic diseases. Nevertheless, many other factors emerged concomitant with the economic growth of China in the past 30 years (e.g., watching television, using a mobile phone and a microwave oven), the effects of which on development of chronic diseases need to be appraised. The effects of genetic factors and the modifying effects of genetic factors on environmental factors on the risk of chronic diseases must also be appraised. Establishing large-scale prospective cohort studies to explore these factors is necessary. In the last decade, large prospective cohort studies have been well underway in Western "developed" countries, but such studies are merely in the primary stage in China.

We aimed to establish a large prospective cohort–Taizhou Longitudinal Study (termed hereafter as the TZL study) in Taizhou (a prefecture of Jiangsu province in China)–to explore the environmental and genetic risk factors for cardiovascular disease, cerebral vascular disease, and cancer. Taizhou is at the junction of north and south China and downstream of the Yangzi River (one of the two largest rivers in China). From the perspective of population size and economic development, Taizhou is a middle-scaled city in China. Taizhou consists of two districts (Hailin and Gaogang) and four county-level cities (Taixing, Xinghua, Jiangyan and Jingjiang). The resident populations for Hailin, Gaogang, Taixing, Xinghua, Jiangyan, and Jingjiang were (in millions) 0.41, 0.18, 1.22, 1.48, 0.86, and 0.66, respectively, according to the fifth national population census of China in 2000 [7]. The Taizhou economy is at the medium level among Chinese cities [8]. Historically, the Taizhou population is a mixture of people from north and south China [9]. Nevertheless, after establishment of the People's Republic of China in 1949, gene flow has been very limited. Taizhou is also well-known for a high prevalence of digestive cancer. According to the surveillance of death causes (1988–2002), the cancer mortality of Taixing was 288.67/100,000 person-years, outclassing the mean for the whole country (108.26/100,000). Among these, esophageal cancer (77.52/100,000), stomach cancer (56.02/100,000) and liver cancer (67.86/100,000) were the commonest malignancies [10].

## Methods

### Study objectives

The TZL study is an open-ended prospective study with very broad research aims. The main objectives of the TZL study are to: (1) describe the mortality and morbidity characteristics of common chronic diseases such as cardiovascular diseases, diabetes mellitus, and cancers; (2) determine environmental risk factors (diet, lifestyle, occupational) and life-course causes of common chronic diseases which are emerging with the economic development of China; (3) determine genetic risk factors underlying common chronic diseases; and (4) determine the contribution of gene-environment interactions to the common chronic diseases.

### Study design

The TZL study was initiated by Fudan University and the Taizhou government in 2007, and "starting seed funding" came from the latter. The TZL study aims to recruit at least 100,000 adults aged 30–80 years from the general population of Taizhou. A three-stage stratified sampling method will be used.

In stage I, one out of three subdistricts (comprising several communities) or towns (comprising several rural communities) will be sampled from Hailing and Gaogang, representing the geographic and economic characteristics in their regions. In stage II, 50% of communities will be randomly selected from each subdistrict or town. In the third stage of sampling, all individuals aged 30–80 years from each household will be chosen.

**Figure 1:**
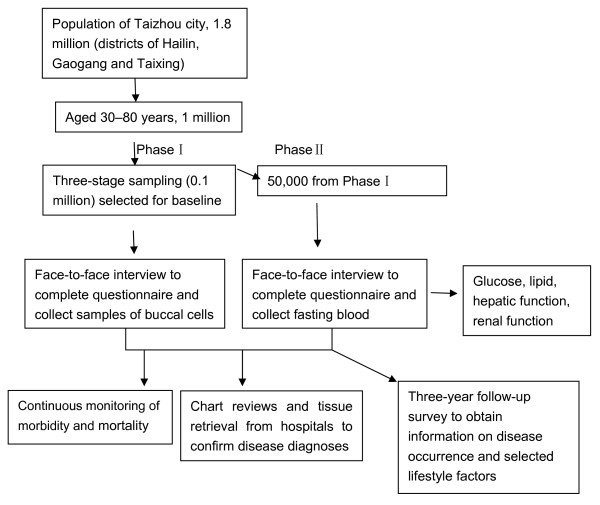
**Subject recruitment, baseline survey, sample collection, and cohort follow-up for the Taizhou Longitudinal Study**.

The TZL study includes a two-phase baseline survey. In phase I, about 100,000 adults aged 30–80 years will be interviewed through questionnaires and samples of buccal mucosal cells will be collected in the communities mentioned above. In phase II, fasting blood samples will be collected for biochemical measurements (e.g., lipid, glucose, hepatic function, renal function) in half of the communities from phase I. After baseline investigation, continuous monitoring of morbidity and mortality will be conducted through a chronic disease register system. Follow-ups will be conducted every three years for event endpoint (see figure 1). The study will use a 'nested' case-control approach for studies on serological markers or genetic biomarkers if sufficient numbers of subjects have developed or died from the particular diseases of interest. By seeking differences in biomarkers in stored DNA and plasma, a wide range of genetic and environmental correlates and causes can be studied. Such an approach allows many factors to be studied in relation to many diseases at relatively low cost.

### Recruitment

A roster of all persons aged 30–80 years was obtained from the offices of the Public Security Bureau, Bureau of Statistics, and Community Committee. Three-to-seven days before the baseline survey, the study staff distributed advertisement material to every household of the target community. A trained interviewer then completed a face-to-face interview. Interviewers were natives who knew the dialect of Taizhou to ensure smooth communication with the participants. After obtaining written informed consent, an in-person interview was conducted using a semi-structural questionnaire to collect baseline data. The interviewer-administered questionnaire covered socioeconomic status, demographic characteristics, residential history, personal habits (e.g., cigarette smoking, alcohol consumption, drinking of tea and coffee), dietary habits (semi-quantitative food frequency questionnaire), family history of selected diseases, cognitive function, physical activity (over the past 5 years and during adolescence), medical history, and, for women only, menstrual/reproductive history and use of hormone therapy. Lifetime occupational history was also obtained in the survey, including all jobs held for at least one year. For each job, the following information was obtained: name of workplace, job title, major products produced or handled, and the period over which each job started and ended. For dietary habits, the questionnaire was designed to capture information on consumption of major food items such as soy foods, allium-type vegetables, and cruciferous and dark-green, leafy vegetables (Table 1). The mean duration of the in-person interview took about 50 minutes.

**Table 1:** Summary of questions covered by the questionnaire at baseline in the Taizhou Longitudinal Study

Exposure category	Variables
Socioeconomic status	Household size, aged ≥ 30 years in household Location of father and mother's birthplace Household income, occupation, education, marriage status
Personal behavior	Smoking, use of computer, mobile phone, and microwave oven, watching of television
Type of food and drink	Drinking of alcohol, tea, and coffee, consumption of food grains, cooking oil, vegetables, meat, poultry, eggs, fresh milk, preserved milk, fish, bean curd,
Cooking style	Fuel (coal, gas, natural gas) Boiled, stir-fried, deep-fried, steamed, grilled, cooked
Environment and occupational exposures	Exposure to chemical solvent, dye, engine oil, gasoline, rubber, plastic product, antiseptic, insecticide, chemical fertilizer, sawdust, asbestos, weaving fiber, dust
Physical activity	Light, middle, and heavy physical labor, physical output in work, during and after work, and leisure time.
Family history	Family history of hypertension, coronary artery disease, diabetes, peptic ulcer, cancer, and other chronic diseases Current age, age at death, and cause of death of first- and second-degree relatives
Medical history	Medical conditions diagnosed by doctors (specifically itemized)
	Use of health services
	Use of Chinese medicines
	Use of Western and other medications
Reproductive history	Age of menarche, menopause status and associated timing, parity and breastfeeding history
	Use of contraceptives
	History of use of hormone-replacement therapy
	Hysterectomy or other gynecological interventions
Psychological status	Stress, anger/hostility, social isolation
Physical examination	Height, weight, waist size, hip size, blood pressure

After the interview, weight, height, waist and hip circumferences, and systolic and diastolic blood pressures were measured. Blood pressure was measured twice by a standardized mercury sphygmomanometer. Systolic blood pressure was recorded to the nearest 2 mmHg at the appearance of the first Korotkoff sound, and diastolic blood pressure was recorded to the nearest 2 mm Hg at the disappearance of the fifth Korotkoff sound. The first reading from each interviewer was discarded, and the second readings from two physicians were recorded and averaged. Weight and height were measured with subjects wearing only light indoor clothing and not wearing shoes. Waist circumference was measured midway between the caudal point of the costal arch as palpated laterally and the iliac crest. Hip circumference was measured at the symphysis-trochanter femoris level. Two measurements were taken, with a tolerance for differences of <1 cm for height, 0.5 cm for circumferences, and 1 kg for weight. A third measurement was taken if the difference between the first two measurements was larger than the defined tolerances.

### Sample collection

After the questionnaire interview and physical measurements, a sample of buccal mucosal cells was collected by mouth rinsing [11]. A blood specimen was drawn after overnight fasting and subjected to centrifugation within three hours and analyzed within eight hours for biochemical markers. Ten milliliters of non-fasting blood were collected into an ethylene diamine tetra-acetic acid (EDTA)-containing vacutainer, a small part of which was used for onsite rapid dipstick testing of random blood glucose and hepatitis-B antigen (HBsAg) before the vacutainer was placed in cool boxes at ~4°C. At the end of each day, blood samples were centrifuged at the local study laboratory and aliquoted into four bar-coded cryovials (three plasma samples and one buffy coat) for long-term storage in nitrogen tanks.

Within about 1–3 weeks of the initial survey, repeat collection of samples is to be obtained on a random sub-sample (3%) of participants, providing estimates of reliability. The Human Ethics Committee of Fudan University approved the study. All information collected is entered directly into a computer using an automated data scan system developed specifically for the project. The data entry program has various built-in functions to avoid missing items and to minimize logic errors and unreasonable responses during the interview.

### Cohort follow-up and outcome ascertainment

Study participants will be followed-up indefinitely by using a chronic disease register system for morbidity and cause-specific mortality already established in Taizhou community-based primary health centers. Nearly all adult deaths in these study areas will undergo some form of investigation, with their underlying causes being certified by a physician. In rare situations (currently 5%) when death occurs at home without recent medical attention, a diagnosis will be conducted by qualified staff based at the regional coordinating center to help determine the most likely cause from symptoms or signs described by family members. Information on non-fatal events will be obtained for certain major categories of diseases (cancer, stroke, and myocardial infarction) through the established registry systems, and by periodically visiting doctors at subdistrict or village health clinics. For suspected cases of non-fatal stroke, coronary heart disease or cancer, further confirmation about their diagnoses will be sought by reviewing records held at hospitals or other medical records.

Although the baseline survey of 100,000 subjects in the study areas is expected to take two years to complete, the mortality follow-up for particular administrative units (such as villages or subdistricts) within each study area will start within six months of the start of the baseline survey in that geographically defined area to allow for the establishment of computerized long-term follow-up systems. The quality and completeness of mortality and morbidity follow-up data in each study area will be checked regularly during the study period. This will involve monitoring the number of people who die or are lost to follow-up each year, as well as assessing overall mortality patterns in the study cohort, levels of diagnostic bases for individual diseases, and the proportion of deaths (in middle age and, separately, in old age) with unknown cause. In addition, a follow-up survey will be conducted every three years to obtain information on disease occurrence and information of selected lifestyle factors. The proportion of deaths with unknown cause or proportion of subjects lost to follow-up should both be 5% (at least up to age 80). It is anticipated that during the first 10 years of follow-up, there will be 6,000 deaths each from cancer and stroke, and 2,000 from coronary heart disease (plus possibly larger numbers of non-fatal events). The substantial size will yield sizeable cases of common cancers (e.g. esophageal carcinoma, gastric cancer, and liver cancer), stroke, and coronary heart disease and generate sample statistical power in estimating the relationship between common exposures where the disease outcome dependent on exposure is also common. For example, assuming a power of 80% and level of significance (two tailed) is 0.05, and assuming a exposure rate of 0.2, the study require 270 case subjects to determine a relative risk of 1.4, or 177 case subjects for a risk of 1.5. .

### Quality assurance and quality control

To ensure implementation and quality of the TZL study, we established a special leadership group, technical advice group, and quality control group. The method of sampling, questionnaire design, training, physical examination, laboratory examinations, and data management have been centralized and standardized. Training of field staff involved in data collection, and office staff handling data entry, checking, and cleaning, has become an established part of our work. Internal controls on quality of measurement are based on collecting measures of selected factors. Interviewer candidates were required to complete standardized training and were certified to conduct independent surveys. Interviews were tape-recorded, and 5% of the tapes were evaluated for interviewing quality. About 3–5% of subjects were re-contacted by phone to evaluate the interviewers' work. Questionnaires and forms were coded twice and were double-entered by different clerks. Inconsistent records were manually checked and corrected. Computer programs were developed to check the logic and reasonable range of responses throughout the questionnaire to identify contradictory responses. In addition to the component-specific quality-control procedures noted above, other procedures were implemented to standardize and monitor the quality of the collection and processing of data. These included training and certification for all interview and examination components, comprehensive manuals of operation, site visits, and blinded duplicate blood measurements (5% sample). The quality and completeness of mortality and morbidity follow-up data in each survey site will be checked regularly during the study period by the study coordinating centers.

## Discussion

The TZL study is focused on exploring the multiple environmental and lifestyles exposures altered during the process of economic transformation of China, solely or in combination with multiple genetic factors, on the gradually increased prevalence of non-communicable chronic diseases. Before 1979, the economy of China was virtually an isolated self-sufficient natural economy. China has since introduced sweeping reforms in the structure of its economy, family planning program, and financial accountability within enterprises and service sector organizations. China simultaneously entered the world economic system. A rapid rise in productivity resulted in continuing increases in the supply of income and food. Ongoing changes on disease patterns and health services accompany the rapid economic transition, as evident from some demographic parameters in the initial pilot phase study conducted from August 2007 to March 2008. This recruited about 17,000 subjects in the Hailin district of Taizhou (Table 2) and is a subject of post-reform China in the new millennium.

**Table 2:** Distribution of selected demographic parameters among subjects recruited in the pilot phase of the Taizhou Longitudinal Study

Exposure	Number of subjects (N = 17314)	Percentage
Use of air conditioning	12412	71.28
Use of microwave	12454	71.52
Use of computer	3832	22.01
Use of mobile phone	3056	17.7
Distance of current house from the main road (m)		
<300	8403	48.25
300–1000 m	4528	26.00
Distance of current house from the factory (m)		
<300	4003	22.99
300–1000	1853	10.64
Moved into a new house during lifetime		
Once	8007	45.98
≥ Twice	1245	7.15

For example, before 1979, being able to use air conditioning, computers, microwave ovens and mobile phones were dreams for Chinese residents, but now about 71%, 22%, 72% and 21%, respectively, of middle-aged and elderly people use these devices. Air pollution and noise pollution accompanied the economic development in China, and became new problems for city residents. About 48% and 26% of residents of Taizhou live in houses <300 m or 300–1000 m from main roads and factories, respectively (Table 2). The influences of these environmental exposures, solely or in combination with genetic factors, on the prevalence of non-communicable chronic diseases require urgent evaluation. With years of follow-up, the TZL study will provide a valuable opportunity to test many important etiologic hypotheses for chronic diseases that cannot be adequately investigated in studies conducted in Western populations.

Large-scale prospective studies are scarce in China, although several large epidemiological surveys have been conducted in China in the last several years. In 1996, a large cohort study, the Shanghai Women's Health Study (SWHS), was initiated to recruit 75,000 women and to collect samples of blood and urine for 20,000 cohort members [12]. Nevertheless, the aim of SWHS was to address some important etiologic hypotheses for cancer and other chronic diseases for women only. The China Health and Nutrition Survey (CHNS) [13] is an ongoing international collaborative project designed to examine the effects of the health, nutrition, and family-planning policies and programs implemented by national and local governments, and to observe how the social and economic transformation of Chinese society is affecting the health and nutritional status of its population. The survey is a multistage, random cluster survey which covers nine provinces that vary substantially in geography, economic development, public resources, and health indicators. The Guangzhou Biobank Cohort Study (GBCS) [14] is an international collaborative study with aims to examine genetic, lifestyle, occupational and environmental factors, and life-course causes of the common chronic diseases that are emerging with economic development. The sample frame of GBCS is members of the Guangzhou Health and Happiness Association for the Respectable Elders (GHHARE) aged ≥ 50 years. These subjects are unlikely to be completely representative of the older population of Guangzhou. For the TZL study, the multistage stratified random cluster sample method guarantees representation of the original population.

The potential weakness of the present study should be stated. First, only buccal mucosal cell samples will be collected in the first phase of our survey, which will include hundreds of thousands of residents. Second, some serological biomarkers cannot be studied. To account for these deficiencies, blood samples will be collected for biological measurement in the second phase. Third, we exclude the possibility of 'healthy volunteer biases' among people who have chosen to join the TZL study. Fourth, similar to all cohorts in "developing" countries, our subjects are increasingly strongly selected survivors with age. In particular, some of our subjects survived childhood infections in a pre-antibiotic environment, and lived through periodic social turmoil up until the 1970s. If survivorship is an issue of concern, we would expect different relationships in older subjects compared with younger subjects, which we will check for routinely. Finally, the present sample size is not sufficient for studying diseases of low incidence, such as specific types of cancer (e.g. prostate cancer).

In the pilot phase of the TZL study, we also encountered some difficulties in field recruitment. For example, the condition of houses of Taizhou residents has improved greatly during the process of economic development. Some of the recruited subjects moving from their old house to a new house now reside in different community. This also caused inconvenience in the pilot phase of the study. We are trying to link our investigation to the removal record of the security bureau of Taizhou district.

In summary, the TZL study will provide a good basis for exploring the roles of many important factors (especially those emerging with the economic transformation of China) in the development of common chronic diseases, solely or via interaction with genetic factors.

## Competing interests

The authors declare that they have no competing interests.

## Authors' contributions

LJ, XFW, and ML designed the study, analyzed the data and drafted the manuscript. JQ, YJY, and SLL organized and participated in the field investigation. DRL, SZY, WM, and WMY were involved the study design and contributed as supervisor and provided all scientific and technical supports. All authors approved the final manuscript.

## Pre-publication history

The pre-publication history for this paper can be accessed here:


